# The Stroke Neuro-Imaging Phenotype Repository: An Open Data Science Platform for Stroke Research

**DOI:** 10.3389/fninf.2021.597708

**Published:** 2021-06-24

**Authors:** Hossein Mohammadian Foroushani, Rajat Dhar, Yasheng Chen, Jenny Gurney, Ali Hamzehloo, Jin-Moo Lee, Daniel S. Marcus

**Affiliations:** ^1^Department of Electrical and System Engineering, School of Engineering, Washington University in St. Louis, St. Louis, MO, United States; ^2^Division of Neurocritical Care, Department of Neurology, Washington University School of Medicine, St. Louis, MO, United States; ^3^Division of Cerebrovascular Disease, Department of Neurology, Washington University School of Medicine, St. Louis, MO, United States; ^4^Department of Radiology, Washington University School of Medicine, St. Louis, MO, United States

**Keywords:** big data, containerized pipeline, deep learning, informatics, phenotype repository, stroke neuroimaging, XNAT

## Abstract

Stroke is one of the leading causes of death and disability worldwide. Reducing this disease burden through drug discovery and evaluation of stroke patient outcomes requires broader characterization of stroke pathophysiology, yet the underlying biologic and genetic factors contributing to outcomes are largely unknown. Remedying this critical knowledge gap requires deeper phenotyping, including large-scale integration of demographic, clinical, genomic, and imaging features. Such big data approaches will be facilitated by developing and running processing pipelines to extract stroke-related phenotypes at large scale. Millions of stroke patients undergo routine brain imaging each year, capturing a rich set of data on stroke-related injury and outcomes. The Stroke Neuroimaging Phenotype Repository (SNIPR) was developed as a multi-center centralized imaging repository of clinical computed tomography (CT) and magnetic resonance imaging (MRI) scans from stroke patients worldwide, based on the open source XNAT imaging informatics platform. The aims of this repository are to: (i) store, manage, process, and facilitate sharing of high-value stroke imaging data sets, (ii) implement containerized automated computational methods to extract image characteristics and disease-specific features from contributed images, (iii) facilitate integration of imaging, genomic, and clinical data to perform large-scale analysis of complications after stroke; and (iv) develop SNIPR as a collaborative platform aimed at both data scientists and clinical investigators. Currently, SNIPR hosts research projects encompassing ischemic and hemorrhagic stroke, with data from 2,246 subjects, and 6,149 imaging sessions from Washington University’s clinical image archive as well as contributions from collaborators in different countries, including Finland, Poland, and Spain. Moreover, we have extended the XNAT data model to include relevant clinical features, including subject demographics, stroke severity (NIH Stroke Scale), stroke subtype (using TOAST classification), and outcome [modified Rankin Scale (mRS)]. Image processing pipelines are deployed on SNIPR using containerized modules, which facilitate replicability at a large scale. The first such pipeline identifies axial brain CT scans from DICOM header data and image data using a meta deep learning scan classifier, registers serial scans to an atlas, segments tissue compartments, and calculates CSF volume. The resulting volume can be used to quantify the progression of cerebral edema after ischemic stroke. SNIPR thus enables the development and validation of pipelines to automatically extract imaging phenotypes and couple them with clinical data with the overarching aim of enabling a broad understanding of stroke progression and outcomes.

## Introduction

Stroke is the second leading cause of death throughout the world, and the leading cause of long-term disability (George et al., 2017). The management of acute stroke is now a time-sensitive emergency that requires organized multidisciplinary care. The early hours after stroke onset frequently map the trajectory of subsequent neurologic disability, complications, and outcomes. Big data analyses can provide an opportunity to implement precision medicine approaches to stroke (Liebeskind, 2018). Pooling of multi-center data sets can advance our understanding of the clinical and biologic factors contributing to outcomes. This has led to a surge of interest and effort to collaborate on stroke research by combining clinical and genomic databases to better understand the biology of stroke and its complications. One of the largest such collaborations [the International Stroke Genetics Consortium (ISGC)] has integrated data on stroke incidence and recovery with genetic data on over 60,000 cases to provide further novel insights into stroke biology (Malik, 2018). There has been special interest in acute stroke phenotypes and outcomes, leading to collaborations within the ISGC and the formation of the Genetics of Neurological Instability after Ischemic Stroke (GENISIS) multi-center study (Heitsch et al., 2021). GENESIS has acquired extensive clinical and genomic data on over 6,000 acute stroke patients.

Brain imaging has a key role in providing further insights about complications after stroke. Indeed, most stroke patients have at least one brain imaging study performed during their acute hospitalization, primarily for diagnostic purposes on presentation. Follow-up scans are often obtained to evaluate the size of infarction and to exclude the development of hemorrhagic transformation. An endeavor is underway to describe the design and rationale for the genetic analysis of acute and chronic cerebrovascular neuroimaging phenotypes detected on clinical magnetic resonance imaging (MRI) in patients with acute ischemic stroke within the scope of the MRI-GENetics Interface Exploration (MRI-GENIE) study (Giese et al., 2017, 2020). Another similar effort with focus on MRI data is Enhancing Neuroimaging Genetics through Meta-Analysis (ENIGMA) Stroke Recovery repository which tries to understand brain and behavior relationships using well-powered meta- and mega-analytic approaches. ENIGMA Stroke Recovery has data from over 2,100 stroke patients collected across 39 research studies and 10 countries around the world (Liew et al., 2020). Although MRI can provide detailed anatomic information, it is challenging to obtain in the acute setting so there is not enough sample which makes knowledge discovery and practical applications limited. Computed tomography (CT) is the most frequently employed modality for acute stroke imaging due to its widespread availability, lower cost, and greater speed of scanning (especially important in acutely unstable patients where “time is brain”) (Tong et al., 2014). Thus, many millions of CT exams of stroke patients with information on stroke location, infarct size, development of edema, and hemorrhagic transformation are available globally. The evaluation of these parameters is not scalable by human raters when leveraging imaging data from thousands of patients. As a result, a big data approach is required to assess images at scale, including identifying quantitative image features and developing automated tools to extract them. This imaging analysis can then be coupled with analysis of clinical and genomics data from these subjects, facilitating large-scale genomic analysis of acute complications after stroke that are best represented by imaging features (Dhar et al., 2018).

Given the potential of imaging to advance our understanding of stroke and its complications, the GENISIS study endeavored to share all clinically performed brain imaging on enrolled subjects. The Stroke Neuroimaging Phenotype Repository (SNIPR) was created as a stroke-focused medical imaging repository that could serve as a platform for this and other stroke-related research. SNIPR is based on the open source XNAT imaging informatics platform, developed at Washington University in St. Louis (WUSTL) (Marcus, 2007). SNIPR provides an environment to securely host and share clinical data and imaging scans from large international stroke cohorts. It also allows the development and deployment of image processing pipelines to extract imaging biomarkers from these stroke scans. SNIPR is deployed on a high-performance computing system that enables these pipelines to be executed as containerized applications at massive scale. SNIPR enables coupling the imaging results with clinical data, with the overarching aim of enabling a broad understanding of stroke progression and outcomes.

## Materials and Methods

**FIGURE 1:**
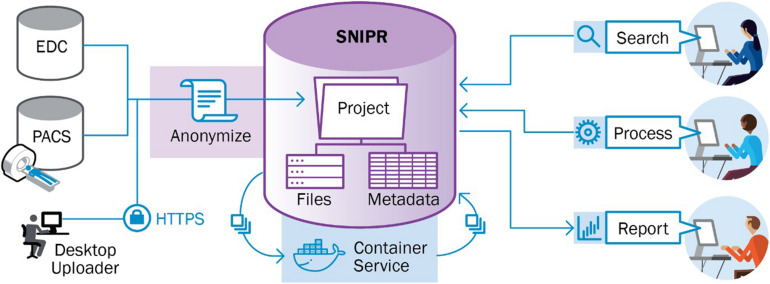
Imaging data can be extracted from picture archiving and communication system (PACS) using DICOM interfaces implemented in XNAT. Alternatively, users can upload DICOM image files using a desktop application. SNIPR automatically removes patient identifying information from DICOM metadata. Clinical attributes can be downloaded from Electronic Data Collection (EDC) systems in spreadsheet format and uploaded into SNIPR via spreadsheets or entered into web-based form. XNAT automatically links the clinical and image data to enable searching and aggregation across domains.

**FIGURE 2:**
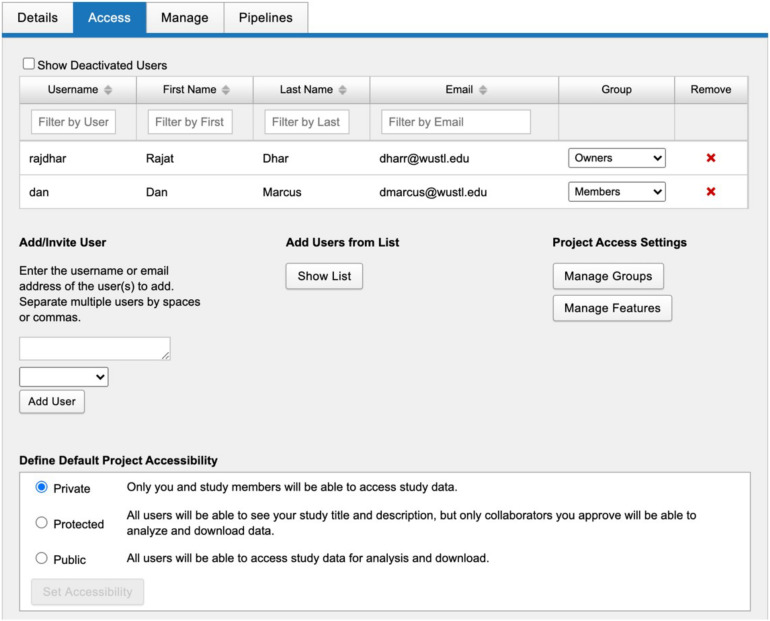
SNIPR uses XNAT’s standard project management mechanism which gives project owners flexible control over access to their data. Projects can be set to public, protected, or private. Protected project data are only accessible to users who have been explicitly granted access to the project.

Stroke Neuroimaging Phenotype Repository was initiated in 2018 to manage the imaging data from the GENISIS multi-center acute stroke genetics study. SNIPR is a modified instantiation of XNAT, an extensible open source imaging informatics platform. The SNIPR image repository is built primarily to support images in DICOM format, the medical imaging industry standard (Pianykh, 2009). Users have multiple paths to importing such images into SNIPR (Figure 1). Exams obtained in the Washington University in St. Louis School of Medicine (WUSTL)-affiliated hospital system can be imported directly from the clinical image repository or Picture Archiving and Communication System (PACS) using XNAT’s PACS query interface, which extracts the images from PACS, anonymizes them, and stores them to the SNIPR file repository. Users can also upload image sessions using the XNAT desktop application, which anonymizes images locally prior to transferring to SNIPR. To guarantee secure transmission of data, outside institutions are restricted to upload through the SNIPR website rather than over the DICOM network protocol, which lacks support for user authentication and authorization. SNIPR’s DICOM anonymization service automatically removes sensitive patient metadata from the file headers following an anonymization profile approved by the WUSTL Institutional Review Board and Department of Radiology Clinical Informatics Section. Once uploaded to SNIPR, relevant DICOM metadata are extracted into the SNIPR database, allowing users to identify and search for data sets via specific acquisition parameters. Each research institution has protected access to its project data, which are only accessible to users who have been explicitly granted access to it. SNIPR uses XNAT’s standard user management, project and data organization, and data access controls. XNAT’s standard project management mechanism provides project owners with flexible control over access to their data (Marcus, 2013; Gurney et al., 2017). Projects can be set to public, protected, or private (Figure 2). Protected project data are only accessible to users who have been explicitly granted access to the project but descriptive project metadata (title, investigator name, and keywords) are accessible to all users. SNIPR can offer a variety of informatics and computational services to support multi-center projects. Access to data can be restricted by site for study coordinators and site investigators, while the overall investigator and administrative core are provided with a pooled view of all the sites’ data. To create projects, upload data, and access non-public data, an individual must create a password- protected user account and login each time they visit the site. Accounts are enabled by system administrators after verification of the submitted credentials. User requests for access to specific data sets are administered by the investigators of the contributed studies, and the data use terms may vary across data sets. SNIPR provides forms for users to request access to data and for investigators to review and approve or reject requests. To further control access, project owners can use the XNAT sharing feature to expose subsets of project data into a secondary project. When users are granted access to a project, they can use a variety of mechanisms to download the data. Imaging sessions can be downloaded as a zip-formatted file or straight to a directory. SNIPR provides a single-session zip downloader, a multi-session bulk downloader through the website, and an interface to download data via a REST programming interface. The multi-session bulk downloader provides support to stop and resume downloads and is able to restart interrupted downloads from previous sessions. All other data in SNIPR such as clinical and image processing results are most typically downloaded by the website into a CSV file. The XNAT REST interface enables users to also download non-imaging data in different formats such as CSV, HTML, XML, and JavaScript Object Notation (JSON). Users can also use the XNAT Advanced Search capability to create their own custom data sets for download.

Additional data types such as imaging and clinical phenotypes can also be added by specifying new data type plugins which are based on XNAT’s standard XML Schema extension mechanism. Indeed, this capability to introduce additional data types empowers SNIPR to support managing multimodal data under a common research subject identifier. When research identifiers are determined by a project owner, then image studies can be directly pulled from PACS and anonymized during import, but clinical data can be downloaded from Electronic Data Collection (EDC) systems in spreadsheet format and uploaded into SNIPR. These anonymized data can be stored under a common research subject identifier in SNIPR so as to support subject level study for various stroke phenotyping at large scale. SNIPR manages subject-level clinical and derived radiological data (Table 1) and session level clinical and derived radiological data (Table 2). The clinical fields in SNIPR reflect measurements widely used in stroke research and are readily expanded to accommodate future studies. These measurements include, but are not limited to, the NIH Stroke Scale/Score (NIHSS) which quantifies stroke severity based on weighted evaluation findings, the Modified Rankin Scale (mRS) which measures degree of disability/dependence after a stroke and the Trial of Org 10172 in Acute Stroke Treatment (TOAST) which denotes five subtypes of ischemic stroke. Assessment of cerebral edema and hemorrhagic transformation after ischemic stroke also follow established grading systems (Larrue et al., 2001; Strbian et al., 2013). Users can use their spreadsheet to upload these clinical data for thousands of subjects at any time and edit these data manually and enter derived radiological data manually after image review.

**TABLE 1:** SNIPR can pull stroke’s clinical data from REDCap and manage these data at subject level.

Subject level EHR	Values
**Entered from image review**	
Lesion location (stroke/ICH)	Cortical/lobar, Subcortical, Both, Lacunar stroke, Cerebellar, Brainstem Acute,
Stroke territory: MCA involved (non-lacunar)	Acute, Subacute only, Chronic only
Stroke territory: ACA involved	Acute, Subacute only, Chronic only
Stroke territory: PCA involved	Acute, Subacute only, Chronic only
Hemorrhagic transformation (ischemic stroke, from all scans)	None, HI-1, HI-2, PH-1, PH-2 (within infarct), Remote PH (or IVH), SAH or SDH
Cerebral edema grading (ischemic stroke)	0 = No edema, 1 = Edema < 1/3 hemisphere (no MLS), 2 = Edema > 2/3 hemisphere (no MLS), 3 = Edema with mid-line shift, 9 = unable to assess
Global cerebral edema (for SAH only)	No, Yes
**Imported from REDCap**	
Disease type	Ischemic stroke, Intracerebral, hemorrhage, Subarachnoid hemorrhage, Traumatic brain injury, Other, no abnormality seen
Modified Rankin Scale (mRS)	0 = No symptoms at all, 1 = No significant disability despite symptoms, 2 = Slight disability, unable to carry out all of previous activities, 3 = Moderate disability, 4 = Moderately severe disability, 5 = Severe disability, 9 = Unknown/Missing data
Trial of Org 10172 in Acute Stroke Treatment (TOAST)	0 = Large artery atherosclerosis, 1 = Cardioembolism, 2- Small vessel disease, 3 = Stroke of other determined etiology, 4 = Stroke of undetermined etiology
NIH Stroke Scale/Score (NIHSS)	0–42
**Imported from REDCap or entered from image review**	
Stroke Side	Right, Left, Both, No stroke seen, Unknown (FU imaging not available at/beyond 24 h)
	

**TABLE 2:** SNIPR can pull stroke’s clinical data from REDCap and manage these data at session level.

Session level EHR	Values
**Entered from image review**	
Hyperdense vessel sign	No, Yes (MCA), Yes (ICA), Yes (Other)
ASPECTS score	1, 2, 3,…, 10, Unable to score, Missing/blank
Infarct present	No, Yes
Midline shift	User defined value in mm
Hemorrhagic transformation	None, HI-1, HI-2, PH-1, PH-2 (within infarct), Remote PH(±
	IVH), SAH or SDH
Intraventricular hemorrhage (for	No, Yes
SAH and ICH)	
**Imported from REDCap or entered from image review**	
Decompressive craniectomy	No, Yes
Ventriculostomy	No, Yes
Old (non-lacunar) stroke present	No, Yes

**FIGURE 3:**
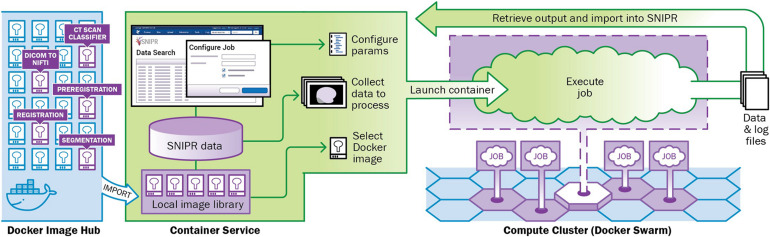
SNIPR is equipped with a container service plugin to manage cluster of containers with Docker swarm on computing cluster attached. Containers used in SNIPR pipelines such as CT scan classifier, DICOM to NIFTI, preregistration, registration, and segmentation are pushed to and pulled from Docker image hub.

Automated imaging data analysis is implemented in SNIPR using XNAT’s Container Service. The Container Service uses a containerized computing architecture to encapsulate applications in portable structures that are readily distributed and deployed to large-scale compute clusters (Di Tommaso et al., 2015; Ismail et al., 2015; Zheng and Thain, 2015; Pienaar et al., 2017). The Container service manages the process of loading container images from public or private repositories (e.g., Docker Hub) to SNIPR’s Docker cluster, provides a user interface for configuring execution parameters and launching container instances to process specific data, and orchestrates execution of containers on the Docker server (Figure 3). SNIPR currently includes containerized applications to perform various tasks such as CT scan type classifier, image format conversion, image pre-registration for skull stripping, longitudinal image-registration, and brain image segmentation. When a containerized application is launched by a user, the container service mounts the necessary data from the SNIPR database in a directory on the container file system from which the containerized application can read and write data. At the conclusion of execution, any files written by the application are exported to the SNIPR database. SNIPR’s automation service can be configured to automatically execute containerized applications when images are uploaded to the database.

**FIGURE 4:**
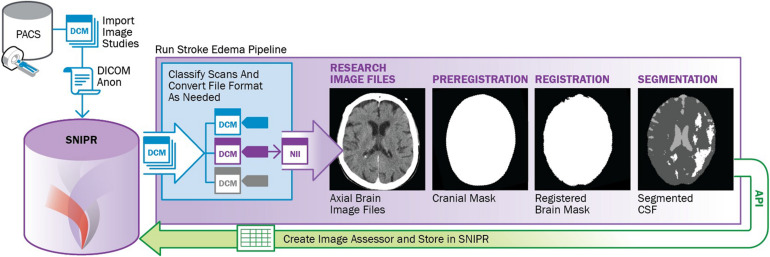
Outline of stroke edema image processing pipeline to analyze CSF volumes from large cohorts of stroke patients.

Stroke Neuroimaging Phenotype Repository supports development and execution of automated and semi-automated processing pipelines. As an example, a containerized stroke edema pipeline was developed to automate image segmentation and measurement of CSF volume in serial CT scans in stroke patients (Figure 4). The pipeline includes five containerized modules, including neural network-based labeling of image acquisition type (Szegedy et al., 2015; Mohammadian Foroushani et al., 2020a), DICOM to NIfTI conversion (Li, 2016), FSL-based preregistration for skull stripping (Jenkinson, 2011), ANTS-based registration of longitudinal brain masks (Avants et al., 2009), U-Net-based segmentation of CSF (Chen et al., 2016), and its volumetric calculation (Dhar et al., 2020). This parallels the recent recommendations for processing head CT data (Muschelli, 2019). After finishing the process, each executed container uses XNAT’s web-based application programming interface (API) to store its output back into SNIPR. The SNIPR user interface provides tools to monitor pipeline progress and review pipeline output, enabling an interactive workflow to check output quality, tune parameters, and relaunch pipeline steps. The pipeline is being used in the large SNIPR patient cohorts to study how dynamic change in brain volumetric values can predict trajectory of edema (Mohammadian Foroushan et al., 2020b).

## Results

**FIGURE 5:**
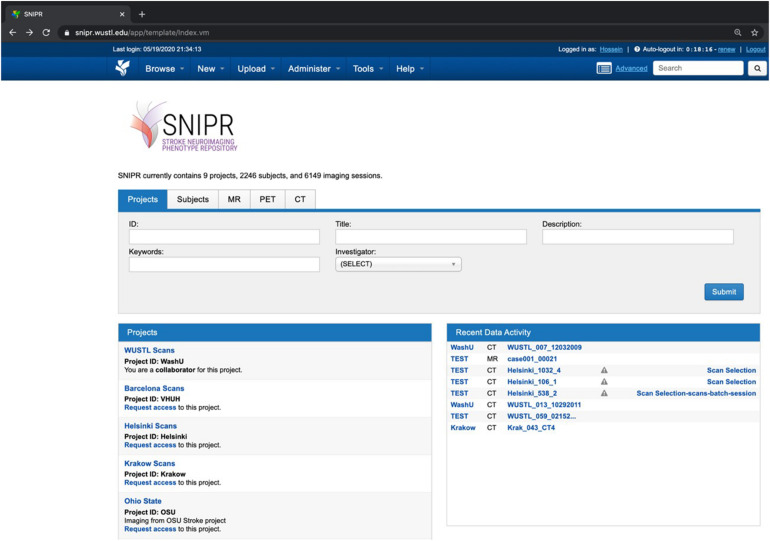
SNIPR hosts eight study cohorts and 2,246 subjects now. SNIPR provides project owners with flexible control over access to their data.

As shown in Figure 5, SNIPR currently hosts nine projects with 2,406 subjects. Of the 6,149 imaging sessions on SNIPR, 6,122 are CT and 85 are MRI. A small number of CT angiography and CT perfusion scans have also been contributed. Each research institution has protected access to its project data, which is only accessible to users who have been explicitly granted access to that project, but all users can see titles of all projects. Table 3 shows summary statistic of all imaging data in SNIPR. Accordingly, SNIPR users have different access levels to projects and SNIPR hosts imaging data of different stroke types such as ischemic, including those with stroke large vessel occlusion and subarachnoid hemorrhage.

**TABLE 3:** SNIPR now manages CT and MR data of 2,246 stroke patients with different disease types such as ischemic stroke, large vessel occlusion stroke, and subarachnoid hemorrhage.

Disease	Projects	Access	Number of study patients	Number of imaging sessions	Imaging modalities
Ischemic stroke	Krakow, Barcelona, Helsinki, WashU,	Private	1,947	4,577	CT, MR
Ischemic stroke-large vessel occlusion	WashU Perfusion, Barnes-Jewish Hospital LVO Strokes, Ohio State	Private	355	873	CT, MR
Subarachnoid	SAH	Private	291	1,526	CT
Hemorrhage					

**FIGURE 6:**
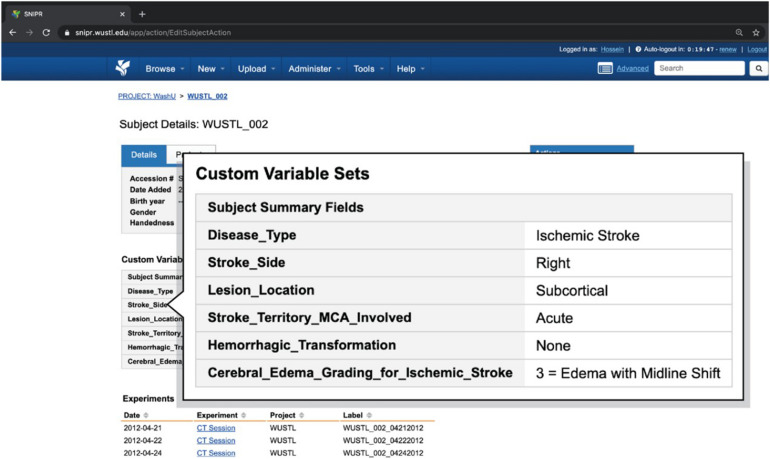
SNIPR can manage clinical data and imaging CT session studies for each subject. Each patient has a subject page where user can have access to patient’s imaging visits and also patient’s clinical data in subject summary table.

**FIGURE 7:**
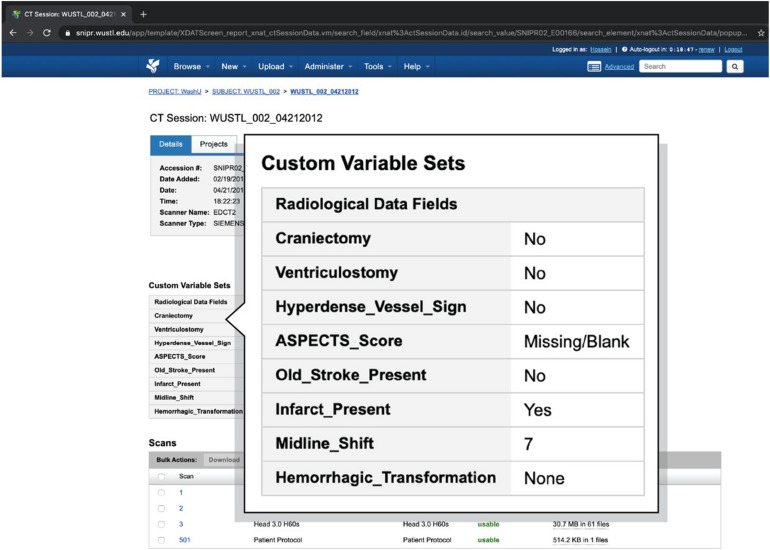
SNIPR can manage clinical data and imaging CT scan studies for all imaging session studies of a patient. Each patient has several visits, each of which has a session page where users can have access to different imaging CT studies and also radiological information particular to that visit.

Web-based reports provide user-friendly access to the demographic, clinical, and imaging attributes captured in SNIPR. As listed in Table 1, researchers can get an overall view of the patient’s stroke complexity such as disease type, stroke side, and lesion location in Subject Summary Field table in patient’s webpages along with having access to their different CT session studies. Figure 6 shows a sample stroke patient whose overall stroke complexity is shown in a table along with list of imaging session studies. So, the researchers can get an overview of stroke complexity for this patient based on this table, and then review imaging sessions for further investigation. As listed in Table 2, SNIPR can also manage session level clinical and derived radiological data. Figure 7 shows a sample session webpage which incorporates various session level clinical and derived radiological data along with each imaging study for this stroke patient. Researchers can get a detailed summary of radiological data such as history of surgery, midline shift, and so on in Radiological Data Fields table and have access to different imaging scans in that specific session study webpage.

## Discussion

SNIPR was developed as a central repository to host and provide secure access to anonymized stroke imaging and associated clinical data, and to serve as a sandbox environment for developing computational pipelines to perform large-scale quantitative analysis. Data type plugins were added to XNAT to extend its support for stroke-related phenotype data type generated by these pipelines and extracted from the electronic medical record. To support scalable computing, advanced containerization tools, including Docker Swarm, were deployed along with a custom Docker scheduling plugin for XNAT. An automated processing pipeline to extract CSF volume in serial CT images was developed and subsequently deployed as a pipeline on SNIPR for use by other projects.

While other repositories such as ENIGMA and MRI-GENIE are critically built with the focus on research MRI with limited longitudinal data, SNIPR offers longitudinal clinical CT (and MRI) which offers much larger numbers and broader representation of available imaging. Also, other repositories have focused on a variety of all neurological and psychiatric disorders or principally on stroke recovery, while SNIPR focuses on stroke-specific data, and it targets acute imaging and relevant acute phenotypes like edema and hemorrhagic transformation. Accordingly, SNIPR can equip researchers to launch processing pipelines at scale to study any stroke phenotype and its trajectory over time to study stroke and its dynamics effectively.

Stroke Neuroimaging Phenotype Repository development is ongoing, with a particular focus on development of additional imaging biomarkers, including predictors of hemorrhagic transformation, measurements of collateral flow, and support of large-scale multicenter clinical studies to integrate different stroke phenotypic studies at massive scale and validate our new discoveries on various populations. The XNAT-based SNIPR database and IT infrastructure will be expanded further to support large-scale data contributions by external collaborators. Data structures will be developed to capture all stroke imaging modalities and multimodal phenotypes. Workflows will be implemented to upload and document data from all data contributors such as anonymization of image metadata, detection and removal of alphanumeric characters in images, and obscuring of identifying facial features. A suite of image quality control pipelines will be implemented to automatically assign quality metrics to uploaded images. A standardized imaging derived disease phenotype, including intracranial compartment, CSF, and infarct volumes, will also be generated for each patient exam and it will be integrated with associated clinical data. New algorithms will be deployed onto SNIPR using XNAT’s Docker container service to enable scalable reproducible processing and validation. A data dashboard will also be implemented as an information management tool that will visually track, integrate, analyze, and display demographic information and processing results for each subject and across all subjects in each project.

## Data Availability Statement

The original contributions presented in the study are included in the article/supplementary material. Further inquiries can be directed to the corresponding author/s.

## Author Contributions

HM drafted the initial manuscript and performed the pipeline development. RD collected the clinical data and revised the manuscript. YC, JG, and AH revised the manuscript. DM and J-ML supervised this project, reviewed the manuscript, and made critical revisions. All authors contributed to the article and approved the submitted version.

## Conflict of Interest

The authors declare that the research was conducted in the absence of any commercial or financial relationships that could be construed as a potential conflict of interest.
